# Effects of Diabetes on Microcirculation and Leukostasis in Retinal and Non-Ocular Tissues: Implications for Diabetic Retinopathy

**DOI:** 10.3390/biom10111583

**Published:** 2020-11-21

**Authors:** Ana Silva Herdade, Iara Mota Silva, Ângelo Calado, Carlota Saldanha, Ngan-Ha Nguyen, Isabella Hou, Miguel Castanho, Sayon Roy

**Affiliations:** 1 Instituto de Medicina Molecular, Faculdade de Medicina, Universidade de Lisboa, Lisboa 1649-004, Portugal; anarmsilva@medicina.ulisboa.pt (A.S.H.); i.motasilva@medicina.ulisboa.pt (I.M.S.); acalado@medicina.ulisboa.pt (Â.C.); carlotasaldanha@gmail.com (C.S.); macastanho@medicina.ulisboa.pt (M.C.); 2 Department of Medicine and Ophthalmology, Boston University School of Medicine, Boston, MA 02118, USA; nganha@bu.edu (N.-H.N.); ihou@bu.edu (I.H.)

**Keywords:** microcirculation, leukostasis, diabetic retinopathy

## Abstract

Changes in retinal microcirculation are associated with the development of diabetic retinopathy (DR). However, it is unclear whether such changes also develop in capillary beds of other non-retinal tissues. Here, we investigated microcirculatory changes involving velocity of rolling neutrophils, adherence of neutrophils, and leukostasis during development of retinal vascular lesions in diabetes in other non-retinal tissues. Intravital microscopy was performed on post-capillary venules of cremaster muscle and ear lobe of mice with severe or moderate diabetes and compared to those of non-diabetic mice. Additionally, number and velocity of rolling leukocytes, number of adherent leukocytes, and areas of leukostasis were quantified, and retinal capillary networks were examined for acellular capillaries (AC) and pericyte loss (PL), two prominent vascular lesions characteristic of DR. The number of adherent neutrophils and areas of leukostasis in the cremaster and ear lobe post-capillary venules of diabetic mice was increased compared to those of non-diabetic mice. Similarly, a significant increase in the number of rolling neutrophils and decrease in their rolling velocities compared to those of non-diabetic control mice were observed and severity of diabetes exacerbated these changes. Understanding diabetes-induced microcirculatory changes in cremaster and ear lobe may provide insight into retinal vascular lesion development in DR.

## 1. Introduction

Diabetic retinopathy (DR) is characterized by progressive microvascular alterations in the retina. The attachment of leukocytes to the luminal endothelium of capillaries, defined as leukostasis, is a prominent cellular event known to promote ischemia and subsequent neovascularization in DR. Studies indicate that increased expression of inflammatory factors, including cell adhesion molecules such as GMP-140, ICAM-1, CD11/CD18 and VCAM-1, promote leukostasis [1,2]. The presence of these molecules expressed on the surface of endothelial cells and leukocytes allows interaction between the two cell types, which could result in attachment of one or more leukocytes [1] leading to non-perfusion and ultimately proliferative DR [2,3,4].

Microcirculation provides steady blood flow to tissues and organs despite changing metabolic conditions and thereby contributes to the maintenance of homeostasis [5,6]. Inadequate microvascular perfusion can lead to adverse effects, such as metabolic acidosis, hyperlactaemia, oxygen depletion, and even organ failure [7]. Thus, most tissues are dependent on active microcirculation to sustain a healthy metabolic microenvironment. In the retina, leukostasis impedes proper functioning of the capillary network, as inflammatory-mediated activation of leukocytes can block retinal capillary microcirculation and drive angiogenesis as a consequence of ischemic conditions [2]. Studies indicate that under severe hyperglycemic condition, leukostasis may increase and contribute to the development of DR [8,9].

Although the mechanisms underlying diabetic microangiopathies remain only partially understood, it is well established that alterations in retinal blood flow could contribute to the development of DR [4,10,11,12,13,14]. Pathophysiological changes in blood flow usually develop gradually in most diseases affecting the microvasculature, including DR [14]. Currently, it is unknown whether microcirculatory properties involving diabetes-induced changes in non-ocular tissues reflect alterations of the retinal microcirculation, including retinal blood flow in the diabetic retina.

Intravital microscopy is a powerful technique for direct visualization and assessment of flow dynamics in small blood vessels. It provides real-time videos of capillaries and allows computational analysis and assessment of blood flow. Changes in microcirculation analyzed through such a visualization technique in sepsis patients correlated with survival of the patients [15].

It seems that monitoring leukostasis could be a useful parameter in providing insight and predicting the severity of retinal vascular lesions in proliferative DR. However, to the best of our knowledge, studies have not specifically addressed leukostasis and its development in non-ocular tissues in the context of DR pathogenesis. In this study, we examined changes in microcirculation in non-ocular tissues (i.e., cremaster muscle and ear lobe) with respect to the number of leukostasis areas in the context of acellular capillaries (AC) and pericyte loss (PL), hallmarks representing early stages of DR, in an animal model of diabetes.

## 2. Materials and Methods

### 2.1. Animals

The animals used in this study received humane care in accordance with the Directive of the European Community 2010/63/EU that mentions the protection of animals used for economic and other scientific ends, and also according to the Portuguese Legislation Law 113/2013. All the proceedings described in this manuscript were approved by the ethical committee of the Animal Welfare Body of Instituto de Medicina Molecular and were submitted to DGAV (Direção Geral de Alimentação e Veterinária) obtaining the legal authorization (protocol number 0421/000/000/2019).

To facilitate visualization of leukocytes, the Lys-EGFP-ki mice were used in this study. This animal model is characterized by the presence of green fluorescent protein (GFP) in the neutrophils. This was achieved by knocking the enhanced GFP (EGFP) gene into the murine lysozyme M (lys) locus [16]. Eighteen Lys-EGFP-ki mice were randomly divided into three groups of six animals each. The first group represents the non-diabetic control mice, the second group represents the low-dose diabetic mice, and the third group the high-dose diabetic mice. Low-dose diabetic mice received streptozotocin (STZ, Sigma-Aldrich, St. Louis, MO, USA) at 40 mg/kg body weight for 5 consecutive days via intraperitoneal (i.p.) injections while the high-dose diabetic mice received STZ at 140 mg/kg body weight in a single dose via an i.p. injection. These are well-established methodologies for inducing diabetes [17].

Fasting blood glucose levels were monitored bi-weekly, and animals with >250–300 mg/dL blood glucose were considered moderately diabetic and those with >300 mg/dL were considered severely diabetic. Insulin was injected to diabetic animals to maintain a steady state glycemic level and avoid severe hyperglycemia. Animals were maintained diabetic for a period of 5 months in an animal facility with a 12 h light/dark cycle and housed in cages in a temperature-controlled room.

Experiments were performed 5 months after the onset of diabetes in STZ-treated diabetic animals. At the end, intravital microscopy of the cremaster muscle and of the ear lobe were performed. Following intravital microscopy, the animals were sacrificed, eyes enucleated, and the retinas subjected to retinal trypsin digestion for isolating the retinal capillary network to assess retinal vascular lesions.

### 2.2. Study Design

Normal (non-diabetic control) and diabetic animals were assessed for number of adherent neutrophils and leukostasis areas in the cremaster muscle and ear lobe using intravital microscopy. Severity of diabetes was taken into consideration in the study design when comparing normal animals to low dose STZ and high dose STZ-induced diabetic animals for the number of rolling leukocytes and rolling velocity in the cremaster and ear lobe microcirculation. In addition, retinal vascular lesions, AC and PL were evaluated in the same animals.

### 2.3. Intravital Microscopy

All animals were kept on a standard mouse diet with food and water ad libitum. For the surgical procedures and microcirculatory measurements, the mice were anesthetized i.p. with a cocktail of xylazine/ketamine (0.1 mL/10 g of BW). Body temperature was maintained between 35 and 37 °C with an auto-regulated heating platform.

The preparation of the ear lobe for intravital microscopy was made by placing the ear of the mice over the lamella of the support and fixing it with silk suture [18]. The preparation of cremaster for intravital microscopy was made in an appropriate support as described in [19]. Using scissors, a small incision in the scrotum was made and one of the testicles was exteriorized. Then the conjunctive tissue that surrounds the cremaster was removed, and an incision in the cremaster was made, fixing it to the appropriate support with silk sutures.

After the cremaster preparation, the support with the animal was placed in a Leica SP8 MP confocal microscope (Leica, Wetzlar, Germany) adapted for intravital microscopy and equipped with a 20× water objective and a 10× lens. All the images were recorded using the LAX 5.0 software. The cremaster was kept in a perfusion of Krebs–Henseleit buffer with NaHCO_3_ warmed to 37 °C and bubbled with 95% N_2_ and 5% CO_2_ for the complete superfusion of the tissue throughout the experiment; excess liquid was removed with a vacuum system. Three different post-capillary venules with 20–25 μm diameter were chosen for the quantification of the parameters, and images were recorded for at least 1 min. From the recorded images, interactions between leukocytes and endothelial cells were quantified for parameters already established [20]: number of rolling leukocytes, their rolling speed, number of adherent leukocytes, and venule diameters. The leukocytes were considered to be rolling on the endothelium if the leukocytes were moving at a slower speed than the erythrocytes in the same vessel over a 1-min duration. The rolling velocity is determined from the time required for a leukocyte to traverse a distance of 100 μm along the length of the venule. A leukocyte was considered adherent to the endothelium if it remained stationary for more than 30 s in a 100 μm length [21]. Leukostasis was determined from images from ear lobe and cremaster capillary networks showing aggregation of leukocytes in localized areas.

### 2.4. Retinal Isolation and Assessment of Acellular Capillaries and Pericyte Loss

To analyze the retinal vasculature for acellular capillaries and pericyte loss, the retinal trypsin digestion (RTD) technique was performed as described previously [22]. Briefly, the retinas were dissected from the enucleated eyes, fixed in 10% formalin, and immersed in 0.5 mol/L glycine buffer for up to 48 h. The retinas were then subjected to 3% trypsin digestion (Becton-Dickinson, San Jose, CA, USA) at 37 °C for approximately 3 h with gentle shaking. Under a dissecting microscope, the nonvascular mass of the retina was removed using a brush (Ted Pella, Redding, CA, USA), and the isolated retinal vascular network mounted onto a silane-coated slide. The capillary networks were then stained with periodic acid Schiff and hematoxylin. Digital images of at least 10 random fields were captured using an Eclipse TE2000-S microscope (Nikon, Tokyo, Japan) attached to a Nikon Digital DS-Fi1 camera and examined for the number of AC and PL.

### 2.5. Statistical Analysis

All data are expressed as mean ± standard deviation (SD). Comparisons between two groups were assessed using a Student’s t-test whereas comparisons between multiple groups (>2 groups) were performed using one-way ANOVA followed by Tukey posthoc multiple comparisons test. The Pearson’s correlation coefficient (R) was calculated to assess the strength of correlation between the number of rolling neutrophils (leukostasis) in the ear lobe and cremaster muscle, and the number of retinal vascular lesions (AC and PL). A level of *p* <0.05 was considered statistically significant.

## 3. Results

The results will present the effects of diabetes on microcirculation in three target tissues (i.e., cremaster, earlobe, and retina). The study focused mainly on microcirculatory changes in the cremaster and ear lobe that were obtained by intravital microscopy and in the retina in which effects of diabetes and those of microcirculatory changes were assessed at the level of retinal vascular lesions (i.e., AC and PL).

### 3.1. Effect of STZ-Induced Diabetes

**Figure 1:**
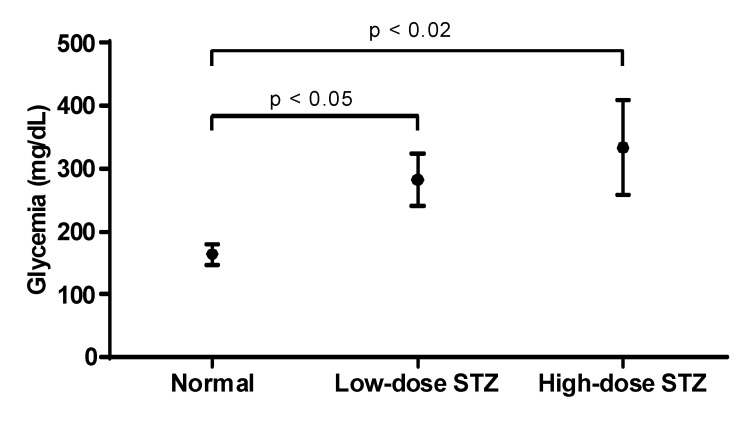
Effect of streptozotocin in mice glucose levels. Mean values (± standard deviation) of glucose levels (mg/dL) measured in normal and diabetic mice (*n* = 6) over a period of 5 months. Two different groups of diabetic mice are presented, the low-dose STZ and the high-dose STZ. Higher doses of streptozotocin yielded higher levels of glycemia.

STZ was injected in mice to induce a low-dose STZ and a high-dose STZ groups. Both groups developed diabetes (Figure 1), but the higher dose of STZ yielded higher levels of glycemia. Mice were considered diabetic when fasting blood glucose exceeded 250 mg/dL for 3 consecutive readings. Based on the severity of hyperglycemia, two mice groups, a moderately diabetic mice group with glycemic values at or below 300 mg/dL and a severely diabetic mice group with glycemic values of over 300 mg/dL were used in the study.

### 3.2. Effect of Diabetes on the Number of Adherent Neutrophils

**Figure 2:**
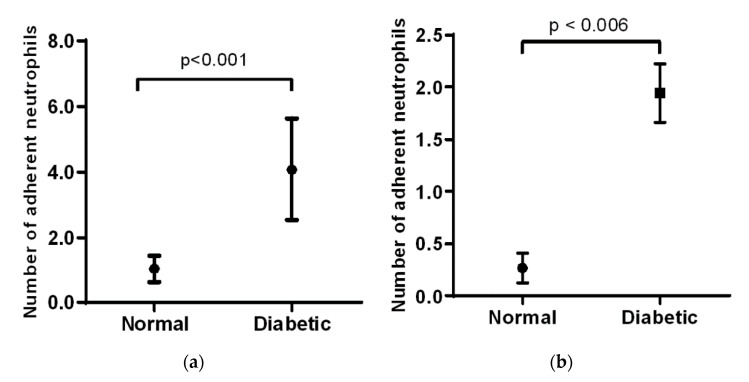
Mean values (± standard deviation) of adherent neutrophils in the cremaster (**a**) and ear lobe (**b**) in normal and diabetic mice (*n* = 6) over a period of 5 months. Overall, diabetic mice exhibited an increased number of adherent neutrophils compared to that of normal mice.

In the cremaster muscle of non-diabetic mice, the number of adherent neutrophils is low, but there is a significant increase in the diabetic mice (1 vs. 4, Figure 2a). In the ear lobe of non-diabetic mice, the number of adherent neutrophils was also low, but this number increases significantly as seen in the diabetic animals (0.25 vs. 2, Figure 2b). When comparing the cremaster muscle and ear lobe of diabetic animals, there were more adherent neutrophils visualized in the cremaster than in the ear lobe.

### 3.3. Effect of Diabetes on the Number of Leukostasis Areas

**Figure 3:**
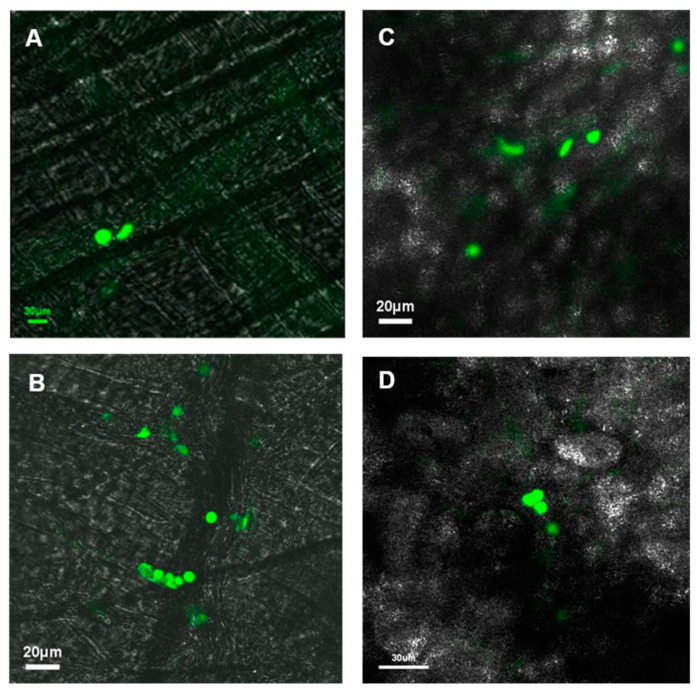

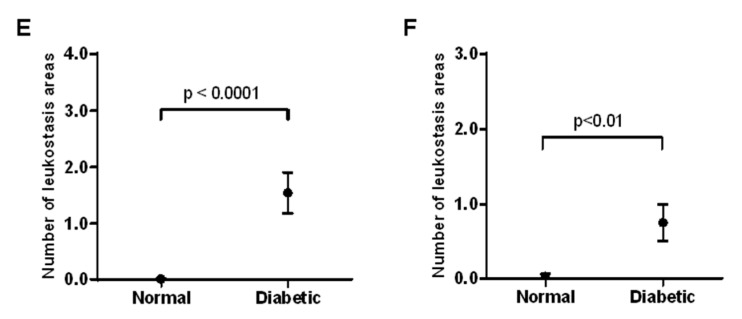
Effect of diabetes on leukostasis. Representative intravital microscopy confocal images of microcirculation showing clustered neutrophils (in green) indicative of leukostasis areas in the cremaster muscle of (**A**) normal and (**B**) diabetic mice, and in the ear lobe of (**C**) normal and (**D**) diabetic mice. Mean values (± standard deviation) of leukostasis areas in the cremaster (**E**) and ear lobe (**F**) in normal and diabetic mice (*n* = 6) over a period of 5 months. Diabetic mice exhibited increased number of leukostasis areas in both cremaster muscle (*p* < 0.0001) and ear lobe (*p* < 0.01) compared to those of normal mice.

In the cremaster muscle of non-diabetic mice, no significant areas of leukostasis were identified, whereas diabetic mice exhibited an increased number of leukostasis areas (Figure 3A). In the ear lobe of non-diabetic mice, no significant areas of leukostasis were identified, but the diabetic mice exhibited an increased number of leukostasis areas (Figure 3B). When comparing the cremaster muscle and ear lobe of diabetic animals, there appeared to be more areas of leukostasis in the cremaster muscle than in the ear lobe.

### 3.4. Effect of Diabetes on the Number of Rolling Neutrophils

**Figure 4:**
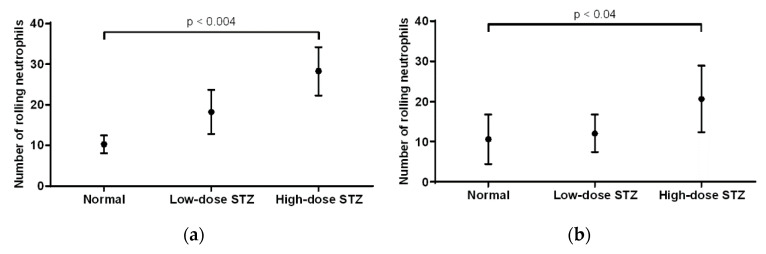
Effects of moderate and severe diabetes in the number of rolling neutrophils. (**a**) Mean values (± standard deviation) of rolling neutrophils in the cremaster muscle of normal and diabetic mice (*n* = 6). Compared to normal mice, mice given high doses of STZ exhibited a significant increase (*p* < 0.004) in the number of rolling neutrophils. Overall, diabetic mice showed an increased number of rolling neutrophils compared to that of normal mice. (**b**) Mean number of rolling neutrophils in the earlobe of normal, low-dose STZ, and high-dose STZ diabetic mice. Compared to normal mice, mice given high dose of STZ exhibited an increase in the number of rolling neutrophils (*p* < 0.04). Overall, diabetic mice showed an increased number of rolling neutrophils compared to that of normal mice.

In the cremaster muscle of non-diabetic mice, the average number of rolling neutrophils was 10 (Figure 4a). Compared to that of non-diabetic mice, the number of neutrophils in mice injected with low or high doses of STZ increased (18 and 28, respectively), with a significant increase (*p* < 0.004) in the severely diabetic mice. Overall, rolling neutrophil counts increased when comparing the cremaster muscle of non-diabetic mice to that of diabetic mice. In the ear lobe of non-diabetic mice, the average number of rolling neutrophils was 10 (Figure 4b). Compared to that of non-diabetic mice, the number of rolling neutrophils of mice injected with low or high doses of STZ increased (12 and 20, respectively), with a significant increase (*p* < 0.04) in the severely diabetic mice. When comparing the cremaster muscle and ear lobe of diabetic animals, there were more rolling neutrophils present in the cremaster than in the ear lobe.

### 3.5. Effect of Diabetes on the Rolling Velocity of Neutrophils

**Figure 5:**
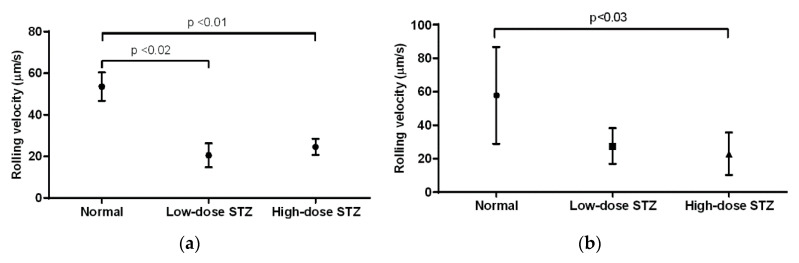
Effects of moderate and severe diabetes in the rolling velocity of neutrophils. (**a**) Mean values of rolling velocity (µm/s) of neutrophils in the cremaster muscle of normal, low-dose STZ, and high-dose STZ diabetic mice (*n* = 6). Compared to normal mice, mice given low doses of STZ exhibited decreased rolling velocity (*p* < 0.02), while mice given higher doses also exhibited decreased rolling velocity (*p* < 0.01). Overall, diabetic mice showed decreased rolling velocity compared to that of normal mice. (**b**) Mean values of rolling velocity (µm/s) in the earlobe of normal, low-dose STZ, and high-dose STZ diabetic mice (*n* = 6). Compared to normal mice, mice given high doses of STZ showed a decreased rolling velocity (*p* < 0.03).

In the cremaster muscle of diabetic mice, the average rolling velocity of neutrophils was measured to be 53 µm/s (Figure 5a). Compared to that of non-diabetic mice, neutrophils of mice injected with low or high doses of STZ exhibited significantly lower rolling velocities (*p* < 0.02 and *p* < 0.01, respectively); however, neutrophils in low-dose animals exhibited lower rolling velocity than neutrophils in high-dose animals (20 vs. 24 µm/s). In the ear lobe of non-diabetic mice, the rolling velocity was measured to be 57 µm/s (Figure 5b). Compared to that of non-diabetic mice, neutrophils of mice injected with low or high doses of STZ exhibited decreased rolling velocities. In particular, neutrophils of mice injected with high doses of STZ showed significantly lower rolling velocity than that of non-diabetic mice (*p* < 0.03). When comparing the cremaster muscle and ear lobe of diabetic animals, we observed similar rolling velocities of the neutrophils.

### 3.6. Effects of Diabetes on the Number of Acellular Capillaries or Pericyte Loss in the Retinal Vasculature

**Figure 6:**
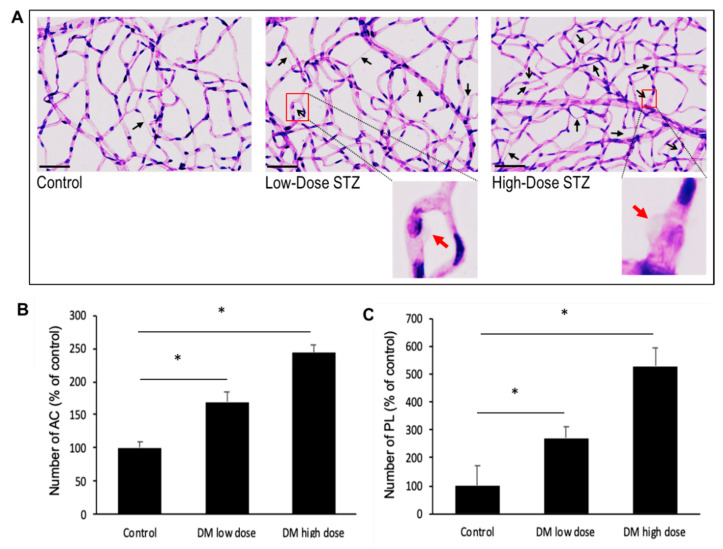
Effects of moderate or severe diabetes on the development of retinal vascular lesions. (**A**) Representative RTD images show increased number of AC (black closed arrows) and PL (black open arrows) in the retinas of moderately diabetic and severely diabetic mice compared to those of control mice (*n* = 6). Scale bar = 100 μm. Enlarged views of pericyte ghosts (red arrows) are shown in boxed areas. (**B**) Graphical illustration of cumulative data shows the number of AC and (**C**) PL progressively increases with diabetes severity. Data are expressed as mean ± standard deviation * = *p* < 0.05.

In this study, we have observed that the diabetic mice exhibit increased number of AC and PL in their retinas compared to those of non-diabetic mice (169 ± 14% of control; *p* < 0.01; 270 ± 42% of control; *p* < 0.02, respectively) (Figure 6A). Importantly, the severely diabetic animals exhibited a higher number of AC (243 ± 12% of control; *p* < 0.01) (Figure 6B) and PL (528 ± 65% of control; *p* < 0.04) (Figure 6C) compared to those of the moderately diabetic animals.

In the ear lobe of high-dose diabetic animals, the number of leukostasis areas compared to the number of retinal vascular lesions showed a strong positive correlation (AC: R = 0.86; *p* = 0.03, PL: R = 0.93; *p* = 0.007). Similarly, in the cremaster muscle of high-dose diabetic animals, the number of leukostasis areas compared to the number of PL showed a strong positive correlation (R = 0.86; *p* = 0.03) but no significant correlation compared to the number of AC (AC: R = 0.774; *p* = 0.06). In parallel, there was no significant correlation between the number of leukostasis areas and the number of retinal vascular lesions in the ear lobe and cremaster of low-dose diabetic animals.

## 4. Discussion

To the best of our knowledge, the findings from this study demonstrate for the first time that diabetes-induced changes in microcirculation develop in the cremaster muscle and ear lobe in parallel with those known to occur in DR. Our results indicate that the four parameters we examined, rolling velocity, number of rolling neutrophils, number of adherent neutrophils, and areas of leukostasis, all seem to worsen with severity of diabetes, in particular, hyperglycemia. The deleterious effects of hyperglycemia have been previously established with respect to the development of diabetic retinopathy. Our current data indicates that changes induced by diabetes in the microcirculation, including the number of areas of leukostasis in the earlobe and cremaster capillary beds, can be detected and that these changes worsen with increased severity of diabetes. Data from this study show a correlation between increase in the number of leukostasis areas in the cremaster and ear lobe versus vascular lesions in the retina of diabetic animals. The extent to which these microcirculatory changes develop in different microvascular beds in the different tissues may vary; however, the data from the current study suggests that these changes develop in parallel in the earlobe, cremaster, and retinal tissues in diabetes.

Microcirculation in different tissues and organs represents a vast capillary network throughout the body. A fundamental function of endothelial cells stems from its characteristic non-thrombogenic surface, which contributes to the maintenance of proper blood flow; however, this could be compromised by dysfunctional endothelium in the diabetic milieu. Endothelial cell functionality may be affected by protein glycation, exposure to pro-inflammatory factors, or by abnormal hemorheological actions [23,24]. It is well established that diabetes affects various functional parameters, including microcirculation in retinal, glomerular, and cremaster muscle capillaries [25]. As such, we examined whether leukocyte-endothelial cell interactions could be affected in the microvascular beds of various tissues and not just in the retina during the pathogenesis of DR [26].

Using intravital microscopy, it was possible to monitor the leukocyte-endothelial cell interactions in the post-capillary venules of the hyperglycemic mice in two different tissues, the cremaster and the ear lobe. Those assessments were performed in the same animal, but the ear lobe images were more difficult, and some even impossible, to count because of the thickness of this tissue. But taking into account all of the parameters that were studied, namely the increased number of adherent neutrophils and the presence of more leukostasis areas accompanied with an increased number of rolling neutrophils and their decreased velocities, we conclude that the observed changes are characteristic of an inflammatory response [27]. To gain deeper insights into our findings regarding diabetes-induced changes in the number of adherent neutrophils and areas of leukostasis, we further examined how severity of diabetes could affect rolling velocity and number of rolling neutrophils. In the cremaster muscle, the number of rolling neutrophils increases, and the rolling velocity decreases significantly in the severely diabetic animals, which promotes a higher number of adherent leukocytes, as described before. In the ear lobe, the same differences were observed with a significant increase in the number of rolling neutrophils and rolling velocities also only in the severely diabetic animals as well. Therefore, what we observed and assessed was the result of a chronic inflammation in the post-capillary venules of diabetic mice. These results are supported by the increase in the number of leukostasis areas seen in both cremaster and ear lobe of diabetic mice compared to those of non-diabetic animals. Leukostasis is known to play an important role in the early phase of the development of DR [2] and therefore can be used as a target for both the early diagnosis and the treatment of this ocular complication. Retinal vascular lesions were also increased in the diabetic animals; an increase in the number of AC and PL were evident, which increased with severity in diabetes.

The presence of retinal pericytes and their integrity are essential for supporting proper functionality of endothelial cells in retinal capillaries [28]. The loss of vascular cells and increase in the number of ACs observed in the retinas of diabetic animals represent structural changes that are known to disturb capillary integrity, impede cell-cell communication through retinal basement membrane, and contribute to diabetic microangiopathy. This type of histological analysis cannot be currently performed in vivo, but advances have been made using imaging techniques via a non-invasive approach. Adaptive optics scanning laser ophthalmoscope (AOSLO) [29] allows direct assessment of cellular structures of the vascular wall, which could be useful as a potential application to identify development and progression of retinal vascular diseases [30]. Findings from the current study provide a framework for a potential application of this non-invasive methodology in diabetic patients for examining structural changes in the retinal capillaries of diabetic individuals.

Microcirculation in the sublingual cavity of patients with sepsis was reported to have a strong correlation with severity of the disease [15]. In our current study, the blood flow disturbance in microcirculation due to leukocyte-endothelial interactions in both the cremaster muscle and ear lobe of diabetic mice also showed a strong similarity with the development of DR. Of note, the accessibility of the earlobe could be advantageous and serve as a surrogate target tissue for capillary imaging and assessments for identifying changes in the microcirculation of the diabetic retina.

## 5. Conclusions

Findings from this study suggest that changes in microcirculation play an important role in the development of diabetic microvascular complications affecting various tissues in the body, including the eyes and the kidneys. Here, we have identified that during diabetes, microcirculation changes in cremaster muscle and ear lobes appear to be correlated and reflect changes in the retinal capillaries in the context of DR. The presence of leukostasis in the ear lobe post-capillary venules represents a specific change seen in both retinal and non-ocular tissue under diabetic conditions. These findings may be useful not only to understand microcirculation changes in different non-ocular tissues in diabetic patients, but also provide a framework for future development of a non-invasive strategy by examining accessible tissues to identify microcirculatory changes occurring in the retinas of diabetic individuals.
